# Don’t get the blues: conspicuous nuptial colouration of male moor frogs (*Rana arvalis*) supports visual mate recognition during scramble competition in large breeding aggregations

**DOI:** 10.1007/s00265-012-1412-6

**Published:** 2012-09-23

**Authors:** Marc Sztatecsny, Doris Preininger, Anita Freudmann, Matthias-Claudio Loretto, Franziska Maier, Walter Hödl

**Affiliations:** 1 Department of Evolutionary Biology, University of Vienna, Althanstrasse 14, 1090 Vienna, Austria; 2 Department of Tropical Ecology and Animal Biodiversity, University of Vienna, Rennweg 14, 1030 Vienna, Austria; 3 Department of Cognitive Biology, University of Vienna, Althanstrasse 14, 1090 Vienna, Austria

**Keywords:** Anuran amphibians, Colour dimorphism, Explosive breeders, Intrasexual selection, Mate recognition, Scramble competition, Visual signals

## Abstract

Conspicuous male colouration is expected to have evolved primarily through selection by female choice. In what way conspicuous colours could be advantageous to males scrambling for mates remains largely unknown. The moor frog (*Rana arvalis*) belongs to the so-called explosive breeders in which spawning period is short; intrasexual competition is strong, and males actively search and scramble for females. During breeding, male body colouration changes from a dull brown (similar to females) to a conspicuous blue, and we wanted to test if male blueness influences mating success or facilitates male mate recognition. To do so, we first measured the colour of mated and non-mated males using a spectrophotometer. In an experiment, we then analysed interactions of actual male moor frogs in natural spawning aggregations with a brown (resembling a female or a non-breeding male) and a blue model frog. Mated and non-mated males did not differ in colouration, suggesting that female choice based on colour traits was unlikely. In our behavioural experiment, male moor frogs spent significantly more time in contact and in amplexus with the brown model than with the blue model. Our results suggest that the nuptial colouration in moor frogs can act as a new type of visual signal in anurans evolved to promote instantaneous mate recognition allowing males to quickly move between rivals while scrambling for females.

**Electronic supplementary material:**

The online version of this article (doi:10.1007/s00265-012-1412-6) contains supplementary material, which is available to authorized users.

## Introduction

Bright colouration and ornaments presented by males during the mating season are a common phenomenon in many animal taxa and are expected to evolve under sexual selection (Andersson and Iwasa 1996). Sexual selection might act through various mechanisms, but female mate choice is most commonly invoked in explaining the evolution of conspicuous male characters. Well-known examples include colour patches in the house finch (Hill 1991), red nuptial colouration in sticklebacks (Milinski and Bakker 1990) and long tail feathers in widowbirds (Andersson 1982). Intrasexual selection through male contests has been assumed to favour the evolution of weapons, such as horns and antlers or signals indicating male condition or fighting ability (Bradbury and Vehrencamp 2011). Examples of male ornaments not related to weapons but rather to enhanced conspicuousness seem less common but have been observed in wolf spiders (Hebets and Uetz 2000) and *Anolis* lizards (Charles and Ord 2012). Another, less-studied mechanism of sexual selection is scramble competition in which males attempt to find a mate before rivals do. Successful males are expected to have well-developed sensory organs and show high mobility (Andersson and Iwasa 1996), but very little is known about colour displays in scrambling species.

Anuran amphibians are well-established model organisms regarding their acoustic communication and role of call features in female choice or male–male agonistic behaviour (Gerhardt and Huber 2002). Understanding parallel issues in colour displays, such as the vocal sac or foot webbings of foot-flagging frog species, has increased during the last decade (Hödl and Amezquita 2001). In the European tree frog, females have been shown to prefer males with bright orange vocal sacs (Gomez et al. 2009), and among competing males of *Micrixalus saxicola* and the Bornean foot-flagging frogs of the genus *Staurois*, the vocal sac and conspicuous foot webbings respectively appear to facilitate the detection of a displaying individual (Grafe et al. 2012; Preininger et al. accepted). Visual cues in anurans with scramble competition belonging to the so-called explosive breeders (Wells 2007) have so far gained less attention, despite the striking colour changes occurring in some species (Ries et al. 2008a; Doucet and Mennill 2010; Sztatecsny et al. 2010). In explosive breeders, all sexually receptive individuals arrive almost synchronously at the spawning site, and breeding takes place over a period of only a few days to weeks. The operational sex ratio (OSR) at spawning sites is generally male biassed, (approximately 50–95 % males, depending on species and population; Wells 2007), and in dense spawning aggregations, males do not call to attract individual females but, instead, search and scramble for access to mates. As females are frequently coerced by males, their options for active choice are limited (but see Hettyey et al. 2009a; Sherman et al. 2010). Still, only about 5 % of the males may breed successfully (Wells 2007). In the struggle to achieve mating success, males, as observed in the common toad (*Bufo bufo*), clasp onto any animate or inanimate object including conspecific males, other amphibian species, dead fish or even beer cans (Reading 1984; Marco and Lizana 2002; MS personal observation). Males clasped by other males perform release calls or vibrations to be released (Wells 2007). Nonetheless, ten or even more common toad males may cling to one female (Verrell and McCabe 1986; Sztatecsny et al. 2006), and after breeding, many drowned females can be found at breeding sites (MS personal observation).

**Fig. 1:**
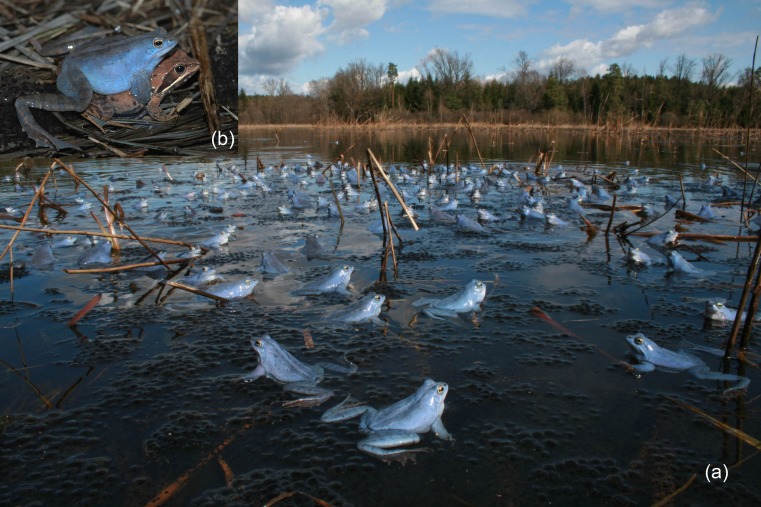
A breeding aggregation of male moor frogs (*R*. *arvalis*) (**a**) and a pair of moor frogs in amplexus (**b**)

The European moor frog (*Rana arvalis*) is a typical explosive breeder but differs from many similar species by the spectacular blue and dynamically changing nuptial colouration of males, which is maintained during the breeding period (Ries et al. 2008a; Fig. 1). This temporal colour dimorphism has been known for more than 100 years (Brehm 1893), but the question of its communicative significance has remained largely unresolved. Male blueness has been reported to be more intense in mated compared to non-mated males (Hedengren 1987; Hettyey et al. 2009c) and to influence offspring survival (Sheldon et al. 2003) but could not be linked to male fertility (Hettyey et al. 2009b). Ries et al. (2008a) measured male colour using a spectrophotometer and found no effect of male body condition or the presence of females on colouration. Considering previous results and moor frog breeding behaviour, we wanted to know if male colouration affects (1) mating success, (2) mate recognition by males or (3) both. To test these hypotheses, we quantified colouration of mated (i.e. in amplexus with a female) and non-mated male moor frogs using spectrophotometry. To experimentally investigate whether body colouration influenced male mating and harassment behaviour, we used a blue and a brown model frog resembling a breeding male and a female or non-breeding male. In support of the first hypothesis, we expected to detect differences in colouration between mated and non-mated males, respectively. In support of the mate-finding hypothesis, we expected to find no colour differences but differences in the response of male moor frogs towards the model frogs, whereas differences in both cases would back up our third hypothesis.

## Methods

### Study species

The moor frog occurs from Central and Eastern Europe through Scandinavia eastwards to Siberia and China (AmphibiaWeb 2012). Our study population belongs to the southern cluster formerly recognised as the subspecies *R*. *arvalis wolterstorffi* (Babik and Rafinski 2000). The dorsal body colouration in both sexes of the moor frog outside the breeding period is generally beige brown, grey and rufous with darker and lighter stripes running along the back. During their migration to the breeding pond, male colouration changes from beige to an almost uniformly dark greyish brown (some males appearing almost pinkish before turning grey). Males turn blue when they enter the pond (Fig. 1; Ries et al. 2008a, b). Upon arriving at the breeding pond, males establish large assemblages of several hundred individuals around suitable communal spawning sites (Fig. 1). The low-intensity advertisement calls (males lack an external vocal sac) are assumed to stimulate females to join the aggregations when they are ready to spawn (Glandt 2006). Male blueness, which is maintained for a few days, and calling activity increase with increasing temperature (Glandt 2006; Hettyey et al. 2009c), and in our study population, both reached the highest intensity during sunny afternoons (MS personal observation). We frequently observed male moor frogs actively searching for approaching females, attempting to take over mated females scrambling among each other. Mating balls of several individuals clinging to a female (Verrell and McCabe 1986; Sztatecsny et al. 2006) seem to be absent (Ries et al. 2008a).

### Frog sampling and spectral reflectance measurements

We collected data for a population of moor frogs at a pond in eastern Austria (47°10′ N, 16°5′ E). As estimated by a spawn clump census, the number of breeding adults exceeded 5,000 individuals in 2011, and spawning took place in dense assemblages where male densities reached 20 individuals m^−2^. In both study seasons (2010 and 2011), spawning activity lasted for 3 days from March 25 to 27. On March 26, 2011, we randomly captured 19 non-mated and 20 mated (in amplexus with a female) males with a dip net from two spawning aggregations and immediately obtained spectral data of each individual to avoid any colour change due to handling. An Ocean Optics Jaz spectrometer with integrated pulsed xenon light source (Jaz-PX) was used to measure spectral reflectance (300–700 nm) at two spots (three measurements per spot): the tympanic membrane (because it is clearly defined) and the flank (because it shows the highest variability in colouration; Ries et al. 2008a). We then measured body mass of each male to the nearest 0.1 g using an electronic miniscale and snout-urostyle length (SUL) to the nearest 0.1 mm using vernier callipers and released all individuals immediately after data collection 100 m away from the spot of capture. From the obtained body measurements, we computed the scaled mass index ${\widehat{M}_i}$ (Peig and Green 2009) as an index of body condition (IBC):

$$ {\widehat{M}_i} = {M_i}{\left[ {\frac{{{L_0}}}{{{L_i}}}} \right]^{{{b_{\mathrm{SMA}}}}}} $$

where *M*_*i*_ and *L*_*i*_ are the body mass and the SUL of individual *i*, respectively; *b*_SMA_ is the scaling exponent estimated by the standardised major axis (SMA) regression of *M* on *L*; *L*_0_ is the mean SUL for our population. The SMA regression was performed with the freeware program SMATR (Falster et al. 2006). Male SUL and IBC have been shown to influence male mating success in explosive breeders and could be indicative of male quality (Davies and Halliday 1977; Höglund and Säterberg 1989; Vásquez and Pfennig 2007; Hettyey et al. 2009d).

### Model frog experiments

#### Model frog design

**Fig. 2:**
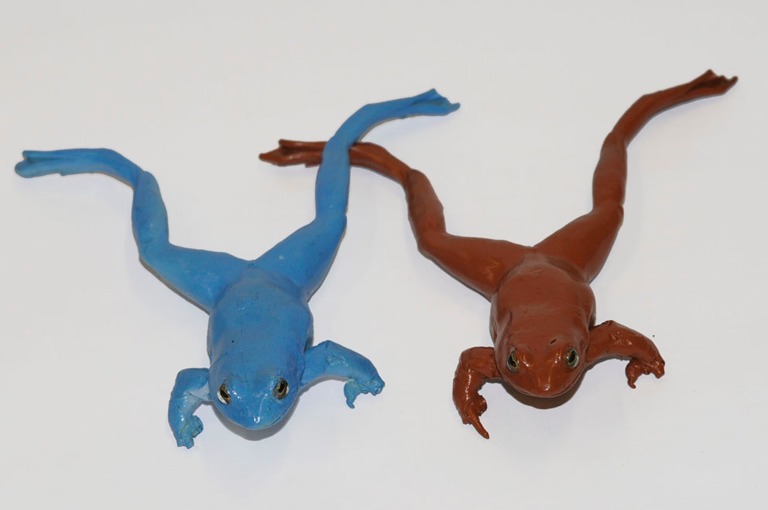
Blue and brown model frogs presented to actual male moor frogs

**Fig. 3:**
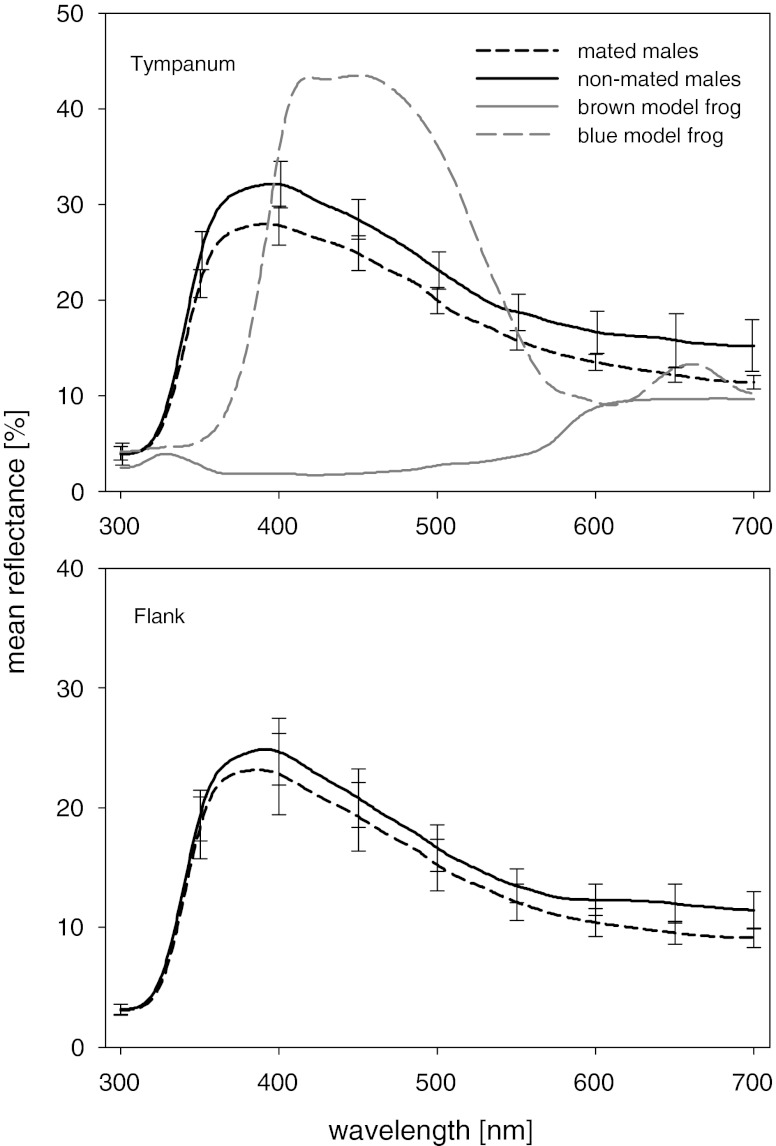
Reflectance spectra of the brown and the blue model frogs and mean reflectance spectra ± SE of the tympanic membrane and the flank of mated (*n* = 20) and non-mated (*n* = 19) male moor frogs

To make our model frogs, we created a silicone cast from a preserved male moor frog specimen and filled it with polyurethane resin (Neukadur MultiCast 1, Altropol, Stocklsdorf, Germany). The resulting cast frogs were fitted with artificial glass eyes and airbrush painted with acrylics in either brown or blue resembling the base colouration of non-breeding males or females and that of breeding males as assessed by the human eye (Fig. 2). Commercial paints absorb in the UV light, resulting in a maximum reflection of the blue model frog at longer wavelengths than in the actual moor frogs (Fig. 3). Finally, a clear coat was sprayed over the models to protect the paint from water and add a realistic sheen. To present the live moor frogs with the models, we attached a blue and a brown model 70 cm apart from each other on fishing rods (1.2 m long) that were connected to the two ends of the cross section of a T-shaped handle. The handle allowed keeping a constant distance between the model frogs and moving both models synchronously with the handler keeping a distance of approximately 1.5 m from models and live frogs.

#### Experiments and data collection

We set up a waterproof camcorder (Panasonic SDR-SW20) on a tripod in spawning aggregations with high densities of male moor frogs. The model frog handler lowered the models into the water and remained still for 5 min for the easily startled frogs to resume natural behaviour. The handler rhythmically moved the T-shaped handle and the attached model frogs a few centimetres up and down (this was necessary because moor frogs would not respond to still models). Interactions between frogs and models were video recorded for 5 min. Subsequently, the position of the model frogs was changed to test different males, and after 2 min of motionlessness (again for the frogs to resume normal activity), the next trial began. We recorded nine trials on 26 March 2010 and six and five trials, respectively, on 26 and 27 March 2011. The videos were analysed with the program Solomon coder (Péter 2011) at a time resolution of 0.30 s. We recoded the time (as the number of 0.30 s intervals) during which there was an active physical contact between frogs and either the blue or the brown model (i.e. pushing with snout and/or fore limbs) or a frog attempted to clasp (amplex) a model.

### Spectral data and statistical analysis

We used the program Avicol v.6 (Gomez 2006) to extract the colour parameters total brightness, hue and UV-blue chroma from our reflectance spectra. Brightness refers to the intensity of the reflectance spectrum (calculated as the area under the spectral curve); hue corresponds to the everyday notion of colour (calculated as the wavelength of maximum reflectance), and chroma, to its saturation or how concentrated the reflectance is around a wavelength (calculated as the reflectance in the interval 300–450 nm divided by total brightness). As we found no differences between mated and non-mated males captured at the spawning assemblages in any of the parameters (i.e. all had turned blue almost equally), we refrained from applying any vision models. To analyse a possible relationship between colour variables and body mass, SUL and IBC of male frogs, we applied Spearman’s correlations. We used a nonparametric test for the experimental data because the variables ‘contact’ and ‘amplexus’ with the blue model were not normally distributed (Shapiro–Wilk test, *P* < 0.05 in both cases). All statistical analyses were performed with Stata/SE 11.0 (StataCorp 2009).

## Results

**Fig. 4:**
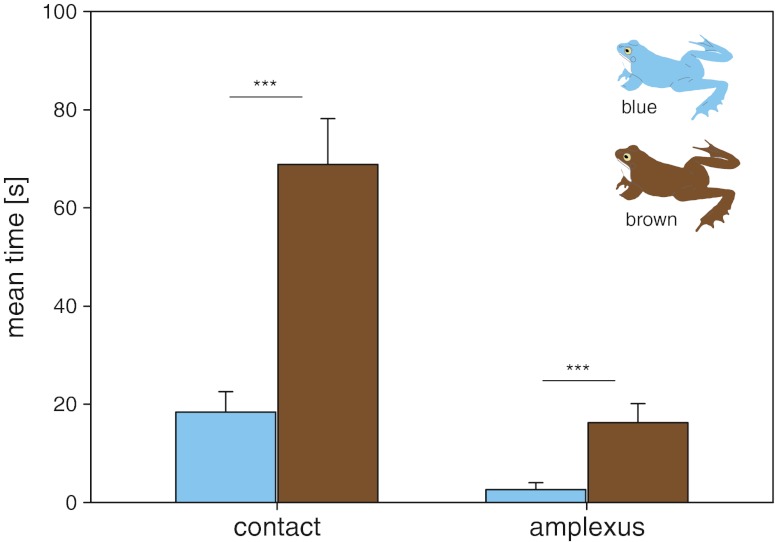
Mean time per trial that male moor frogs spend in active contact or in amplexus with a blue model frog resembling the colour of a breeding male and a brown model frog resembling a non-breeding male (and also the colouration of females)

Mated male moor frogs differed neither significantly in the colouration variables brightness, hue and chroma (Table 1, Fig. 3) nor in SUL, body mass or IBC from their non-mated rivals. Average SUL ± standard error (SE) of the 19 non-mated and the 20 mated males was 56.96 ± 1.13 and 56.57 ± 0.8 mm; mean body mass ± SE was 27.28 ± 1.77 and 25.26 ± 1.49 g, and mean ${\widehat{M}_i}$ ± SE was 27.15 ± 1.67 and 25.23 ± 1.42, respectively (Wilcoxon signed-rank test, *P* > 0.1 for all comparisons). We also found no significant correlation between colour variables and male frogs’ body mass, SUL and ${\widehat{M}_i}$ (Spearman correlation, *P* > 0.07 in all cases). In the behavioural experiments, male moor frogs spent almost four times as much time in contact and in amplexus with the brown as with the blue frog model with the differences being highly significant (Wilcoxon signed-rank test; contact, *z* = 3.008, *n* = 20, *P* < 0.001; amplexus, *z* = 3.17, *n* = 20, *P* < 0.002; Fig. 4).

**Table 1:** Comparison of body colouration parameters of non-mated and mated male moor frogs

	Non-mated males	Mated males	*Z*	*P*
	Mean ± SE	Mean ± SE		
Flank				
Total brightness	6,241.35 ± 632.78	5,602.49 ± 739.43	0.87	0.38
Hue	418.84 ± 21.72	414.5 ± 21.93	1.28	0.20
Blue chroma	0.43 ± 0.01	0.43 ± 0.01	−0.08	0.93
Eardrum				
Total brightness	8,354.4 ± 718.06	7,089.03 ± 476.24	1.64	0.10
Hue	404.16 ± 8.2	391.2 ± 2.98	0.66	0.51
Blue chroma	0.42 ± 0.02	0.44 ± 0.003	0.16	0.88

## Discussion

Our study showed that mating success in male moor frogs was not significantly influenced by colouration. Males, however, were able to discriminate model frogs and favoured the brown model, similar in colour to a female or a non-breeding male compared to the blue model, resembling a rival breeding male. We therefore argue that the nuptial colouration of male moor frogs is unlikely to indicate male condition or to be subject to female mate choice. By increasing a male’s conspicuousness and its contrast to females, the blue colouration can act as a new type of visual signal directed at other males evolved to promote swift mate recognition and mate finding in dense aggregations. Among competing individuals, a signal has been suggested to remain reliable when competitors at least partially share an interest in common (Lachmann and Bergstrom 1998; Szamado 2011). All male moor frogs are rivals while trying to find a female, but it should be in the interest of all males not to miss a mating opportunity and to reduce mate-searching effort because females are limited (Johnstone et al. 1996; Loman and Madsen 2010). Mate-searching behaviour might be energetically demanding for males of explosive breeders, as they can lose up to 1 % of their total body mass/day during the reproductive period (Arak 1983; Ryser 1989). A conspicuous visual signal making breeding males distinguishable from females and non-breeding males can reduce mate-searching time by (1) facilitating the visual detection of females, (2) minimising unwanted clasping attempts with rivals and (3) reducing harassment by rivals. A male not expressing the blue body colouration would be subject to frequent mating attempts by other males and would only be released after emitting a release call (Liao and Lu 2009; Chen and Lu 2011). Release calls emitted by male moor frogs have a significantly lower mean sound pressure level (34 dB, *n* = 9 males) at 1-m distance compared to calls of the common toad (46 dB, *n* = 12 males), in which males do not differ from females in body colour (*t* test, *t* = 6.78, DF = 19, *P* < 0.001, Preininger unpublished data). A visual signal may function more rapidly than calls allowing individuals to communicate instantaneously and therefore move more quickly among rivals while scrambling for mates. Colour displays reducing male mating attempts are known from female lizards signalling non-receptivity (Chan et al. 2009) and female damselflies mimicking male body colouration in order to reduce male harassment in species with strong sexual conflict (Van Gossum et al. 2011; Xu and Fincke 2011).

Surprisingly, mated males in our study population did not differ significantly from non-mated males in body size, mass or IBC, traits that should be advantageous during direct male–male competition (Arak 1983). A number of previous studies on explosive breeders found large male advantage (e.g. Davies and Halliday 1977; Hedengren 1987; Wells 2007 and references therein), whereas others failed to do so (e.g. Höglund and Robertson 1987; Wells 2007 and references therein; Greene and Funk 2009; Hettyey et al. 2009c). The observed discrepancies in study outcomes indicate that mating systems of explosive breeders are dynamic, with mating behaviour likely varying with population size, individual density and time available for competition (Höglund and Robertson 1988; Höglund 1989; Wells 2007). It also has been suggested that mate-searching tactics in small males differ from those of large males, with large males being more successful in achieving takeovers of females in direct competition with already mated males (Arak 1983; Höglund and Robertson 1988). Small males may profit from higher mobility compared to large males as indicated in males of scrambling insect species (Moya-Larano et al. 2007; Kelly et al. 2008) and attempt to find approaching females on the edge of breeding aggregations.

The actual process of colour change in male moor frogs has not yet been investigated, but it is assumed that blue colour in amphibians derives from incoherent light scattering from reflecting platelets in specific chromatophores, the iridophores (Bagnara et al. 2007). Shifts in the adjacent chromatophore layers of yellow xanthophores and dark melanophores lead to rapid colour change. The dependence of blueness on temperature (Hettyey et al. 2009c), which should be linked to a general level of activity in ectotherms and the ability to change colouration, suggests that male blueness could be adjusted to the intensity of intrasexual competition. Traits involved in male–male competition have been shown to be influenced by population density and OSR (Knell 2009; Bretman et al. 2011). For instance in dung beetles, males from species with low-density populations and a male-biassed OSR develop horns used as weapons for intrasexual aggressive behaviour (Pomfret and Knell 2008). In species with high population density and female excess, males scramble for matings and lack horns enhancing their mobility and ability to find females. The social environment males that are confronted with may not only vary between but also within species, and males may respond with behavioural plasticity by choosing alternative mating strategies (Bretman et al. 2011). For instance, male common toads have been shown to switch from chorusing behaviour at low densities to active searching and scrambling when the number of breeding individuals increases (Höglund and Robertson 1988). Blueness of male moor frogs might also vary in response to the social environment and would be predicted to be most intense in large, high-density populations such as our study population.

Sexual dimorphism in colour or pattern is known to occur in only 25 anuran species (Hoffman and Blouin 2000), but male nuptial colour change in explosive breeders has been reported for at least ten species from six families (Supplementary Material), emphasising the importance of body colouration as a social signal in this group. Explosive breeding species showing temporal colour dimorphism could therefore be an ideal study system to better understand flexible sex roles and signal plasticity in variable ecological and social environments.

## Electronic supplementary material

Below is the link to the electronic supplementary material.

ESM 1 (DOC 43 kb)
