# Supplementary material for: Don’t get the blues: conspicuous nuptial colouration of male moor frogs (Rana arvalis) supports visual mate recognition during scramble competition in large breeding aggregations
Source: Behav Ecol Sociobiol. 2012 Sep 23;66(12):1587–93. doi: 10.1007/s00265-012-1412-6 (PMC3496481; doi:10.1007/s00265-012-1412-6)
Supplement: Supplementary file 1 — (DOC 43 kb) [file 265_2012_1412_MOESM1_ESM.doc]

**Don’t get the blues: conspicuous nuptial colouration of male moor frogs (*Rana arvalis*) supports visual mate recognition during scramble competition in large breeding aggregations**

M. Sztatecsny*, D. Preininger, A. Freudmann, M-C. Loretto, F. Maier, W. Hödl

Department of Evolutionary Biology, University of Vienna, Althanstrasse 14, 1090 Vienna

*Author for correspondence: [marc.sztatecsny@univie.ac.at](mailto:marc.sztatecsny@univie.ac.at)

**Species list of known explosively breeding anurans with temporal colour change**

| **Species** | **Family** | **Range** | **male nuptial colour** | **references** |
| --- | --- | --- | --- | --- |
| *Amietophrynus (Bufo) kisoloensis* | Bufonidae | Eastern Africa | yellow | AmphibiaWeb |
| *Amietophrynus (Bufo) maculatus* | Bufonidae | Sub-Saharan Africa | yellow | Rödel 2000 |
| *Amietophrynus (Bufo) togoensis* | Bufonidae | West Africa | yellow (not all males!) | Rödel unpubl. data |
| *Duttaphrynus (Bufo) melanostictus* | Bufonidae | India, South-East Asia | yellow | Daniels 2005 |
| *Incilius (Bufo) luetkenii* | Bufonidae | Central America | yellow | Doucet & Mennill 2010 |
| *Hoplobatrachus tigerinus* | Dicroglossidae | India, Madagascar (introduced) | yellow | Daniels 2005, Glaw & Vences 2007 |
| *Osteocephalus leprieurii* | Hylidae | South America | yellow | Hödl pers. obs. |
| *Aglyptodactylus madagascariensis* | Mantellidae | Madagascar | yellow | Glaw & Vences 2007 |
| *Phrynobatrachus alleni* | Phrynobatrachidae | Western West Africa | yellow | Rödel 2003 |
| *Rana arvalis* | Ranidae | Europe, Northern Asia | blue | Ries et al. 2008 |
| *R. temporaria* | Ranidae | Europe, Northern Asia | white throat | Sztatecsny et al. 2010 |

**References:**

AmphibiaWeb: Information on amphibian biology and conservation. 2011 Berkeley, California: AmphibiaWeb Available: http://amphibiaweb.org

Daniels R.J.R. 2005 Amphibians of Peninsular India. Hyderabad, University Press.

Doucet S.M., Mennill D.J. 2010 Dynamic sexual dichromatism in an explosively breeding Neotropical toad. Biology Letters 6(1), 63-66. (doi:10.1098/rsbl.2009.0604).

Hödl pers. obs.

Glaw F., Vences M. 2007 Field Guide to the Amphibians and Reptiles of Madagascar. Köln, Vences and Glaw Verlag.

Ries C., Spaethe J., Sztatecsny M., Strondl C., Hödl W. 2008 Turning blue and ultraviolet: Sex-specific colour change during mating season in the Balkan Moor Frog. Journal of Zoology 276, 229-236.

Rödel, M.-O. (2000): Herpetofauna of West Africa, Vol. I: Amphibians of the West African savanna. – Edition Chimaira, Frankfurt/M., 335 pp.

Rödel, M.-O. (2003): The amphibians of Mont Sangbé National Park, Ivory Coast. – Salamandra, **39**: 91-110.

Sztatecsny M., Strondl C., Baierl A., Ries C., Hödl W. 2010 Chin up: are the bright throats of male common frogs a condition-independent visual cue? Anim Behav 79, 779-786.
